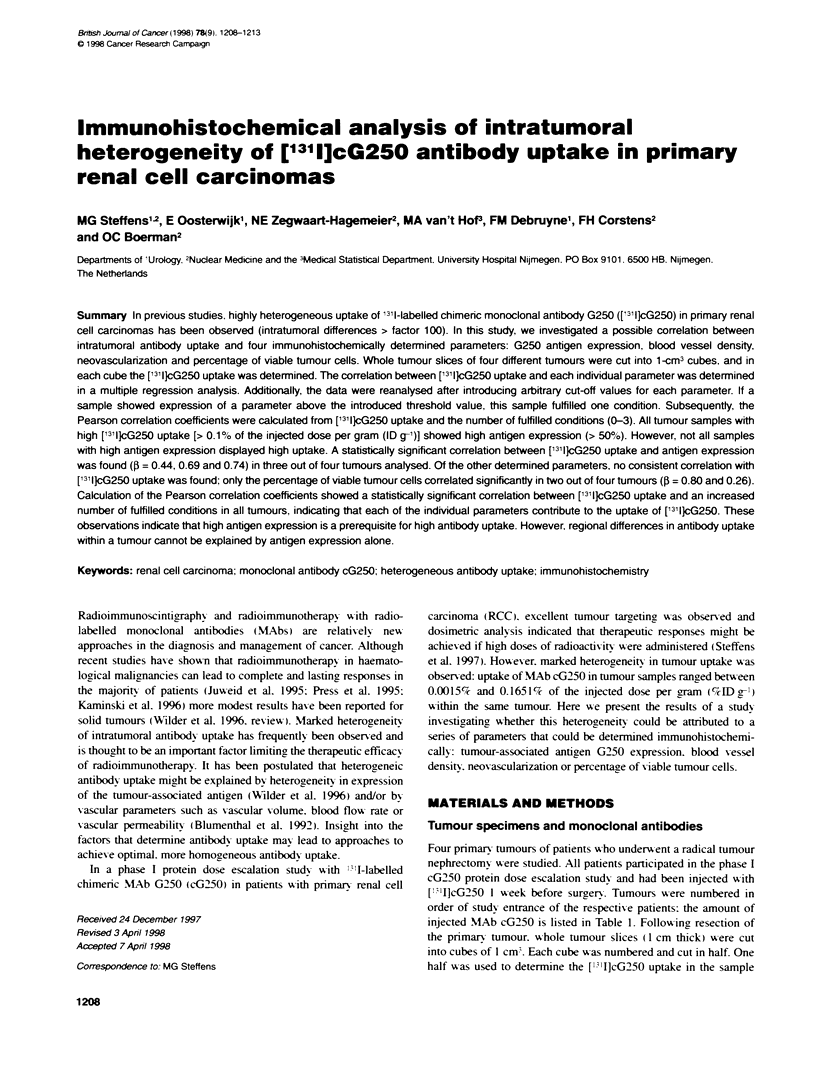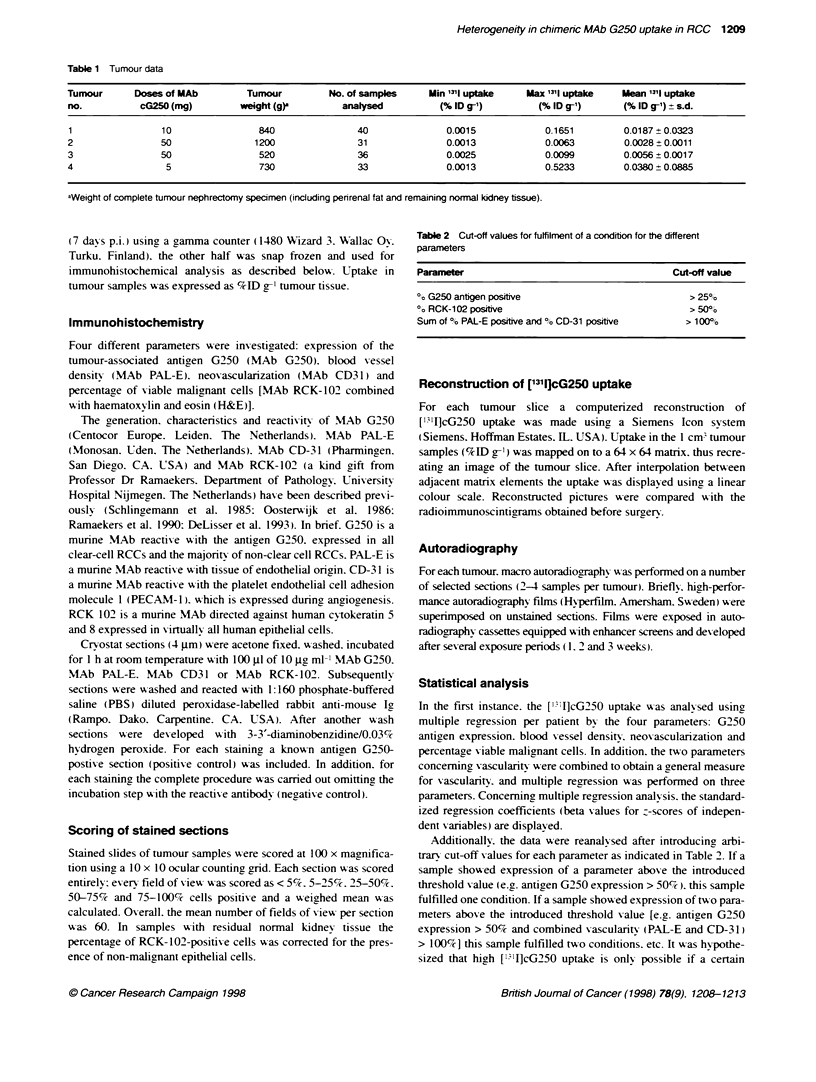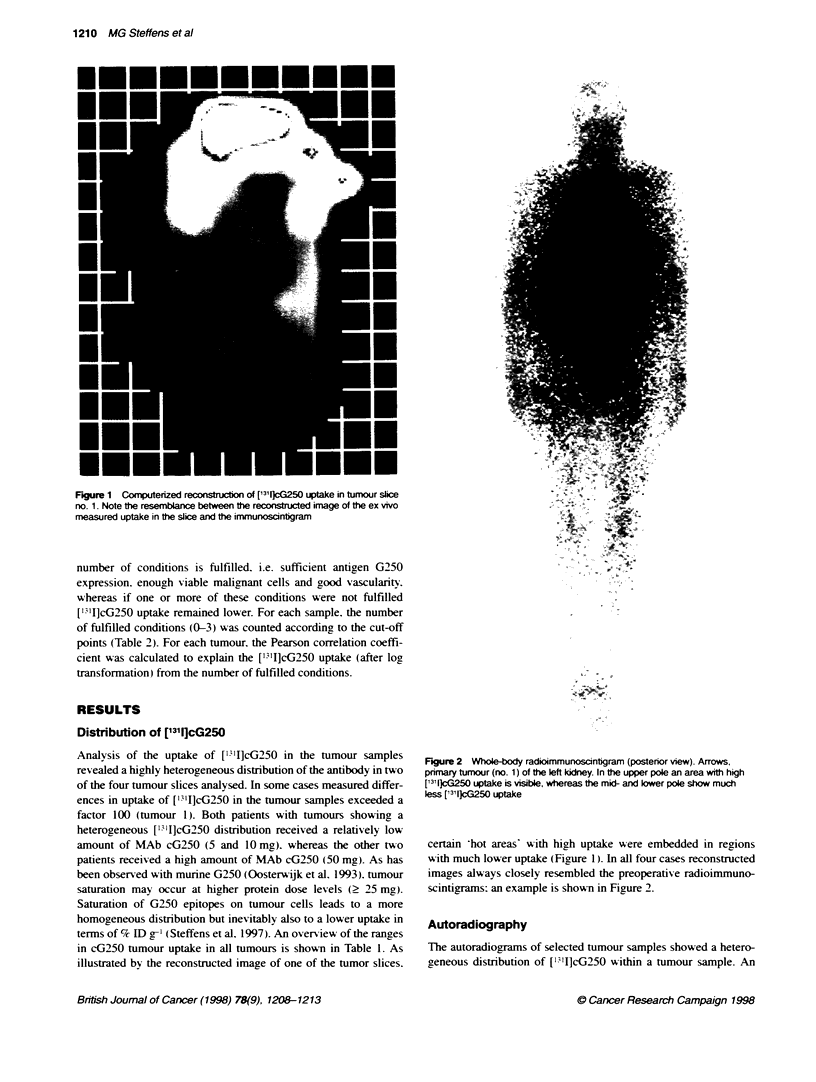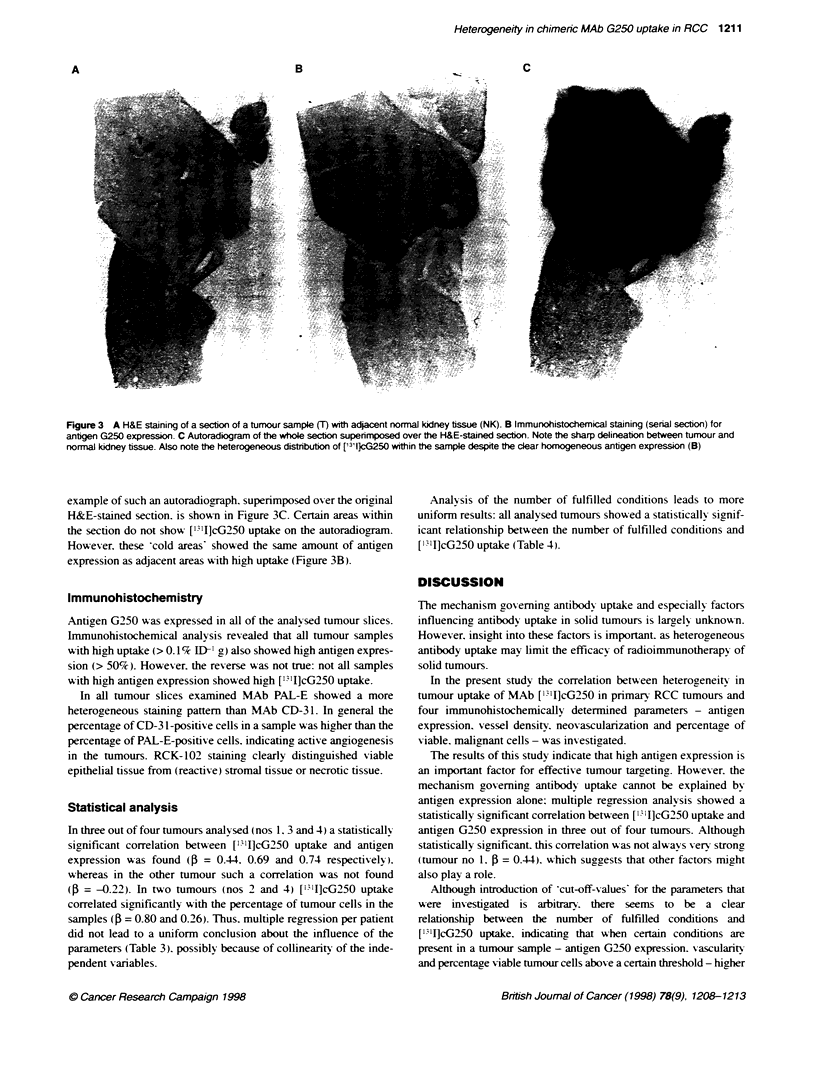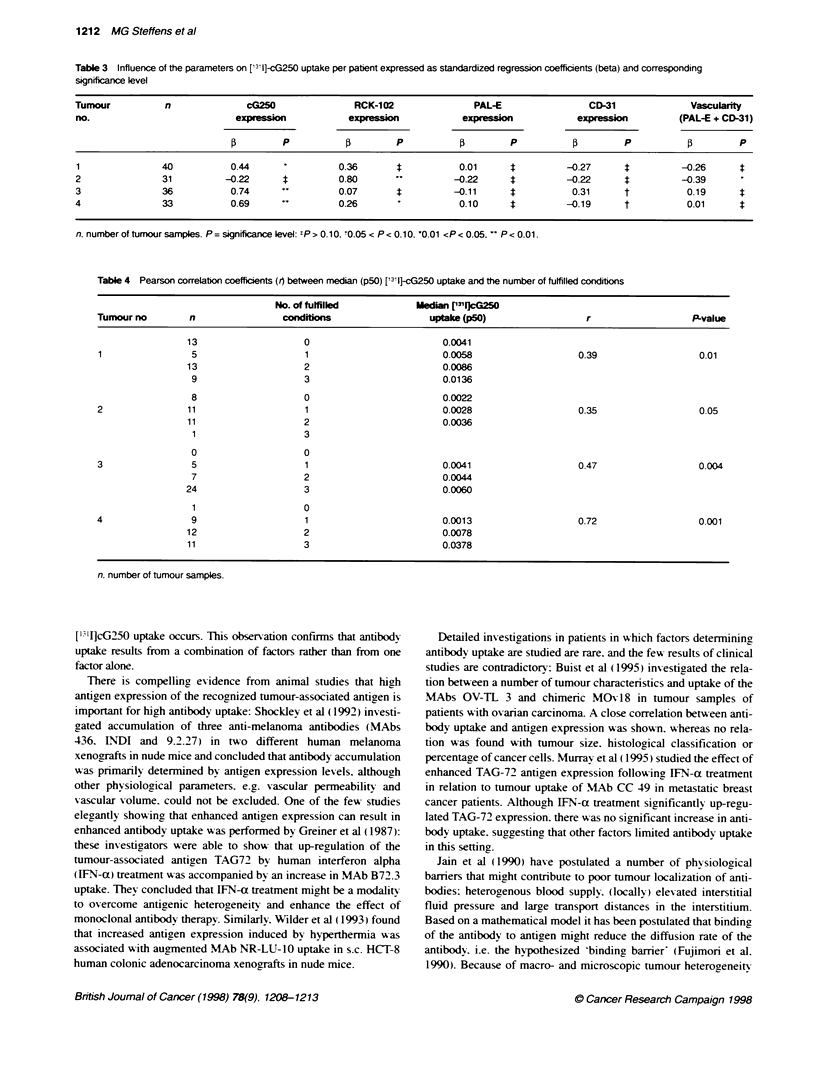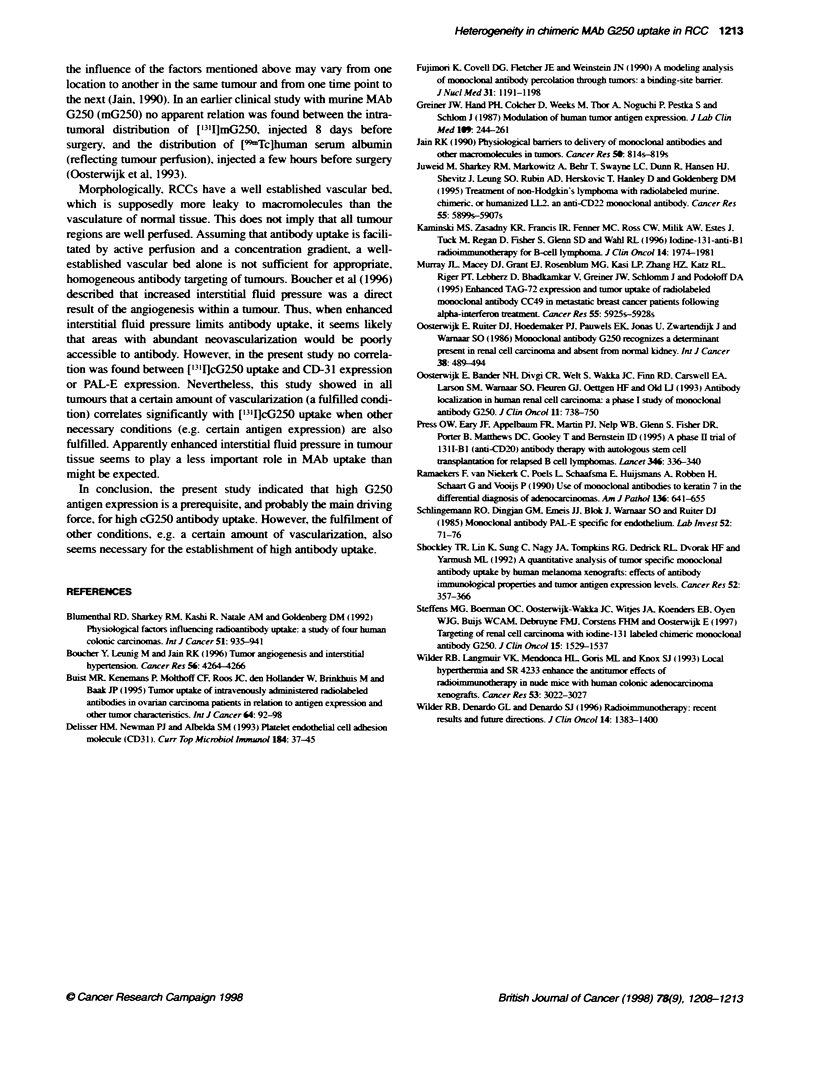# Immunohistochemical analysis of intratumoral heterogeneity of [131I]cG250 antibody uptake in primary renal cell carcinomas.

**DOI:** 10.1038/bjc.1998.656

**Published:** 1998-11

**Authors:** M. G. Steffens, E. Oosterwijk, N. E. Zegwaart-Hagemeier, M. A. van't Hof, F. M. Debruyne, F. H. Corstens, O. C. Boerman

**Affiliations:** Department of Urology, University Hospital Nijmegen, The Netherlands.

## Abstract

**Images:**


British Journal of Cancer (1998) 78(9), 1208–1213
© 1998 Cancer Research Campaign

### Immunohistochemical analysis of intratumoral heterogeneity of [$^{131}$I]cG250 antibody uptake in primary renal cell carcinomas

MG Steffens[1,2], E Oosterwijk[1], NE Zegwaart-Hagemeier[2], MA van't Hof[3], FM Debruyne[1], FH Corstens[2] and OC Boerman[2]

Departments of [1]Urology, [2]Nuclear Medicine and the [3]Medical Statistical Department, University Hospital Nijmegen, PO Box 9101, 6500 HB, Nijmegen, The Netherlands

**Summary** In previous studies, highly heterogeneous uptake of $^{131}$I-labelled chimeric monoclonal antibody G250 ([$^{131}$I]cG250) in primary renal cell carcinomas has been observed (intratumoral differences > factor 100). In this study, we investigated a possible correlation between intratumoral antibody uptake and four immunohistochemically determined parameters: G250 antigen expression, blood vessel density, neovascularization and percentage of viable tumour cells. Whole tumour slices of four different tumours were cut into 1-cm$^3$ cubes, and in each cube the [$^{131}$I]cG250 uptake was determined. The correlation between [$^{131}$I]cG250 uptake and each individual parameter was determined in a multiple regression analysis. Additionally, the data were reanalysed after introducing arbitrary cut-off values for each parameter. If a sample showed expression of a parameter above the introduced threshold value, this sample fulfilled one condition. Subsequently, the Pearson correlation coefficients were calculated from [$^{131}$I]cG250 uptake and the number of fulfilled conditions (0–3). All tumour samples with high [$^{131}$I]cG250 uptake [> 0.1% of the injected dose per gram (ID g$^{-1}$)] showed high antigen expression (> 50%). However, not all samples with high antigen expression displayed high uptake. A statistically significant correlation between [$^{131}$I]cG250 uptake and antigen expression was found ($\beta$ = 0.44, 0.69 and 0.74) in three out of four tumours analysed. Of the other determined parameters, no consistent correlation with [$^{131}$I]cG250 uptake was found; only the percentage of viable tumour cells correlated significantly in two out of four tumours ($\beta$ = 0.80 and 0.26). Calculation of the Pearson correlation coefficients showed a statistically significant correlation between [$^{131}$I]cG250 uptake and an increased number of fulfilled conditions in all tumours, indicating that each of the individual parameters contribute to the uptake of [$^{131}$I]cG250. These observations indicate that high antigen expression is a prerequisite for high antibody uptake. However, regional differences in antibody uptake within a tumour cannot be explained by antigen expression alone.

**Keywords:** renal cell carcinoma; monoclonal antibody cG250; heterogeneous antibody uptake; immunohistochemistry

Radioimmunoscintigraphy and radioimmunotherapy with radiolabelled monoclonal antibodies (MAbs) are relatively new approaches in the diagnosis and management of cancer. Although recent studies have shown that radioimmunotherapy in haematological malignancies can lead to complete and lasting responses in the majority of patients (Juweid et al, 1995; Press et al, 1995; Kaminski et al, 1996) more modest results have been reported for solid tumours (Wilder et al, 1996, review). Marked heterogeneity of intratumoral antibody uptake has frequently been observed and is thought to be an important factor limiting the therapeutic efficacy of radioimmunotherapy. It has been postulated that heterogeneic antibody uptake might be explained by heterogeneity in expression of the tumour-associated antigen (Wilder et al, 1996) and/or by vascular parameters such as vascular volume, blood flow rate or vascular permeability (Blumenthal et al, 1992). Insight into the factors that determine antibody uptake may lead to approaches to achieve optimal, more homogeneous antibody uptake.

In a phase I protein dose escalation study with $^{131}$I-labelled chimeric MAb G250 (cG250) in patients with primary renal cell carcinoma (RCC), excellent tumour targeting was observed and dosimetric analysis indicated that therapeutic responses might be achieved if high doses of radioactivity were administered (Steffens et al, 1997). However, marked heterogeneity in tumour uptake was observed: uptake of MAb cG250 in tumour samples ranged between 0.0015% and 0.1651% of the injected dose per gram (%ID g$^{-1}$) within the same tumour. Here we present the results of a study investigating whether this heterogeneity could be attributed to a series of parameters that could be determined immunohistochemically: tumour-associated antigen G250 expression, blood vessel density, neovascularization or percentage of viable tumour cells.

*Received 24 December 1997*

*Revised 3 April 1998*

*Accepted 7 April 1998*

*Correspondence to:* MG Steffens

### MATERIALS AND METHODS

### Tumour specimens and monoclonal antibodies

Four primary tumours of patients who underwent a radical tumour nephrectomy were studied. All patients participated in the phase I cG250 protein dose escalation study and had been injected with [$^{131}$I]cG250 1 week before surgery. Tumours were numbered in order of study entrance of the respective patients; the amount of injected MAb cG250 is listed in Table 1. Following resection of the primary tumour, whole tumour slices (1 cm thick) were cut into cubes of 1 cm$^3$. Each cube was numbered and cut in half. One half was used to determine the [$^{131}$I]cG250 uptake in the sample

**Table 1** Tumour data

| Tumour no. | Doses of MAb cG250 (mg) | Tumour weight (g)[a] | No. of samples analysed | Min $^{131}$I uptake (% ID $g^{-1}$) | Max $^{131}$I uptake (% ID $g^{-1}$) | Mean $^{131}$I uptake (% ID $g^{-1}$) ± s.d. |
|---|---|---|---|---|---|---|
| 1 | 10 | 840 | 40 | 0.0015 | 0.1651 | 0.0187 ± 0.0323 |
| 2 | 50 | 1200 | 31 | 0.0013 | 0.0063 | 0.0028 ± 0.0011 |
| 3 | 50 | 520 | 36 | 0.0025 | 0.0099 | 0.0056 ± 0.0017 |
| 4 | 5 | 730 | 33 | 0.0013 | 0.5233 | 0.0380 ± 0.0885 |

[a]Weight of complete tumour nephrectomy specimen (including perirenal fat and remaining normal kidney tissue).

(7 days p.i.) using a gamma counter (1480 Wizard 3, Wallac Oy, Turku, Finland), the other half was snap frozen and used for immunohistochemical analysis as described below. Uptake in tumour samples was expressed as %ID $g^{-1}$ tumour tissue.

### Immunohistochemistry

Four different parameters were investigated: expression of the tumour-associated antigen G250 (MAb G250), blood vessel density (MAb PAL-E), neovascularization (MAb CD31) and percentage of viable malignant cells [MAb RCK-102 combined with haematoxylin and eosin (H&E)].

The generation, characteristics and reactivity of MAb G250 (Centocor Europe, Leiden, The Netherlands), MAb PAL-E (Monosan, Uden, The Netherlands), MAb CD-31 (Pharmingen, San Diego, CA, USA) and MAb RCK-102 (a kind gift from Professor Dr Ramaekers, Department of Pathology, University Hospital Nijmegen, The Netherlands) have been described previously (Schlingemann et al, 1985; Oosterwijk et al, 1986; Ramaekers et al, 1990; DeLisser et al, 1993). In brief, G250 is a murine MAb reactive with the antigen G250, expressed in all clear-cell RCCs and the majority of non-clear cell RCCs. PAL-E is a murine MAb reactive with tissue of endothelial origin. CD-31 is a murine MAb reactive with the platelet endothelial cell adhesion molecule 1 (PECAM-1), which is expressed during angiogenesis. RCK 102 is a murine MAb directed against human cytokeratin 5 and 8 expressed in virtually all human epithelial cells.

Cryostat sections (4 µm) were acetone fixed, washed, incubated for 1 h at room temperature with 100 µl of 10 µg $ml^{-1}$ MAb G250, MAb PAL-E, MAb CD31 or MAb RCK-102. Subsequently sections were washed and reacted with 1:160 phosphate-buffered saline (PBS) diluted peroxidase-labelled rabbit anti-mouse Ig (Rampo, Dako, Carpentine, CA, USA). After another wash sections were developed with 3-3′-diaminobenzidine/0.03% hydrogen peroxide. For each staining a known antigen G250-postive section (positive control) was included. In addition, for each staining the complete procedure was carried out omitting the incubation step with the reactive antibody (negative control).

### Scoring of stained sections

Stained slides of tumour samples were scored at 100 × magnification using a 10 × 10 ocular counting grid. Each section was scored entirely: every field of view was scored as < 5%, 5–25%, 25–50%, 50–75% and 75–100% cells positive and a weighed mean was calculated. Overall, the mean number of fields of view per section was 60. In samples with residual normal kidney tissue the percentage of RCK-102-positive cells was corrected for the presence of non-malignant epithelial cells.

**Table 2** Cut-off values for fulfilment of a condition for the different parameters

| Parameter | Cut-off value |
|---|---|
| % G250 antigen positive | > 25% |
| % RCK-102 positive | > 50% |
| Sum of % PAL-E positive and % CD-31 positive | > 100% |

### Reconstruction of [$^{131}$I]cG250 uptake

For each tumour slice a computerized reconstruction of [$^{131}$I]cG250 uptake was made using a Siemens Icon system (Siemens, Hoffman Estates, IL, USA). Uptake in the 1 $cm^3$ tumour samples (%ID $g^{-1}$) was mapped on to a 64 × 64 matrix, thus recreating an image of the tumour slice. After interpolation between adjacent matrix elements the uptake was displayed using a linear colour scale. Reconstructed pictures were compared with the radioimmunoscintigrams obtained before surgery.

### Autoradiography

For each tumour, macro autoradiography was performed on a number of selected sections (2–4 samples per tumour). Briefly, high-performance autoradiography films (Hyperfilm, Amersham, Sweden) were superimposed on unstained sections. Films were exposed in autoradiography cassettes equipped with enhancer screens and developed after several exposure periods (1, 2 and 3 weeks).

### Statistical analysis

In the first instance, the [$^{131}$I]cG250 uptake was analysed using multiple regression per patient by the four parameters: G250 antigen expression, blood vessel density, neovascularization and percentage viable malignant cells. In addition, the two parameters concerning vascularity were combined to obtain a general measure for vascularity, and multiple regression was performed on three parameters. Concerning multiple regression analysis, the standardized regression coefficients (beta values for $z$-scores of independent variables) are displayed.

Additionally, the data were reanalysed after introducing arbitrary cut-off values for each parameter as indicated in Table 2. If a sample showed expression of a parameter above the introduced threshold value (e.g. antigen G250 expression > 50%), this sample fulfilled one condition. If a sample showed expression of two parameters above the introduced threshold value [e.g. antigen G250 expression > 50% and combined vascularity (PAL-E and CD-31) > 100%] this sample fulfilled two conditions, etc. It was hypothesized that high [$^{131}$I]cG250 uptake is only possible if a certain

© Cancer Research Campaign 1998

**Figure 1** Computerized reconstruction of [$^{131}$I]cG250 uptake in tumour slice no. 1. Note the resemblance between the reconstructed image of the ex vivo measured uptake in the slice and the immunoscintigram number of conditions is fulfilled, i.e. sufficient antigen G250 expression, enough viable malignant cells and good vascularity, whereas if one or more of these conditions were not fulfilled [$^{131}$I]cG250 uptake remained lower. For each sample, the number of fulfilled conditions (0–3) was counted according to the cut-off points (Table 2). For each tumour, the Pearson correlation coefficient was calculated to explain the [$^{131}$I]cG250 uptake (after log transformation) from the number of fulfilled conditions.

### RESULTS

### Distribution of [$^{131}$I]cG250

Analysis of the uptake of [$^{131}$I]cG250 in the tumour samples revealed a highly heterogeneous distribution of the antibody in two of the four tumour slices analysed. In some cases measured differences in uptake of [$^{131}$I]cG250 in the tumour samples exceeded a factor 100 (tumour 1). Both patients with tumours showing a heterogeneous [$^{131}$I]cG250 distribution received a relatively low amount of MAb cG250 (5 and 10 mg), whereas the other two patients received a high amount of MAb cG250 (50 mg). As has been observed with murine G250 (Oosterwijk et al, 1993), tumour saturation may occur at higher protein dose levels (≥ 25 mg). Saturation of G250 epitopes on tumour cells leads to a more homogeneous distribution but inevitably also to a lower uptake in terms of % ID g$^{-1}$ (Steffens et al, 1997). An overview of the ranges in cG250 tumour uptake in all tumours is shown in Table 1. As illustrated by the reconstructed image of one of the tumor slices, certain 'hot areas' with high uptake were embedded in regions with much lower uptake (Figure 1). In all four cases reconstructed images always closely resembled the preoperative radioimmunoscintigrams; an example is shown in Figure 2.

**Figure 2** Whole-body radioimmunoscintigram (posterior view). Arrows, primary tumour (no. 1) of the left kidney. In the upper pole an area with high [$^{131}$I]cG250 uptake is visible, whereas the mid- and lower pole show much less [$^{131}$I]cG250 uptake

### Autoradiography

The autoradiograms of selected tumour samples showed a heterogeneous distribution of [$^{131}$I]cG250 within a tumour sample. An

© Cancer Research Campaign 1998

**Figure 3** **A** H&E staining of a section of a tumour sample (T) with adjacent normal kidney tissue (NK). **B** Immunohistochemical staining (serial section) for antigen G250 expression. **C** Autoradiogram of the whole section superimposed over the H&E-stained section. Note the sharp delineation between tumour and normal kidney tissue. Also note the heterogeneous distribution of [$^{131}$I]cG250 within the sample despite the clear homogeneous antigen expression (**B**)

example of such an autoradiograph, superimposed over the original H&E-stained section, is shown in Figure 3C. Certain areas within the section do not show [$^{131}$I]cG250 uptake on the autoradiogram. However, these 'cold areas' showed the same amount of antigen expression as adjacent areas with high uptake (Figure 3B).

### Immunohistochemistry

Antigen G250 was expressed in all of the analysed tumour slices. Immunohistochemical analysis revealed that all tumour samples with high uptake (> 0.1% ID$^{-1}$ g) also showed high antigen expression (> 50%). However, the reverse was not true: not all samples with high antigen expression showed high [$^{131}$I]cG250 uptake.

In all tumour slices examined MAb PAL-E showed a more heterogeneous staining pattern than MAb CD-31. In general the percentage of CD-31-positive cells in a sample was higher than the percentage of PAL-E-positive cells, indicating active angiogenesis in the tumours. RCK-102 staining clearly distinguished viable epithelial tissue from (reactive) stromal tissue or necrotic tissue.

### Statistical analysis

In three out of four tumours analysed (nos 1, 3 and 4) a statistically significant correlation between [$^{131}$I]cG250 uptake and antigen expression was found ($\beta$ = 0.44, 0.69 and 0.74 respectively), whereas in the other tumour such a correlation was not found ($\beta$ = –0.22). In two tumours (nos 2 and 4) [$^{131}$I]cG250 uptake correlated significantly with the percentage of tumour cells in the samples ($\beta$ = 0.80 and 0.26). Thus, multiple regression per patient did not lead to a uniform conclusion about the influence of the parameters (Table 3), possibly because of collinearity of the independent variables.

Analysis of the number of fulfilled conditions leads to more uniform results: all analysed tumours showed a statistically significant relationship between the number of fulfilled conditions and [$^{131}$I]cG250 uptake (Table 4).

### DISCUSSION

The mechanism governing antibody uptake and especially factors influencing antibody uptake in solid tumours is largely unknown. However, insight into these factors is important, as heterogeneous antibody uptake may limit the efficacy of radioimmunotherapy of solid tumours.

In the present study the correlation between heterogeneity in tumour uptake of MAb [$^{131}$I]cG250 in primary RCC tumours and four immunohistochemically determined parameters – antigen expression, vessel density, neovascularization and percentage of viable, malignant cells – was investigated.

The results of this study indicate that high antigen expression is an important factor for effective tumour targeting. However, the mechanism governing antibody uptake cannot be explained by antigen expression alone; multiple regression analysis showed a statistically significant correlation between [$^{131}$I]cG250 uptake and antigen G250 expression in three out of four tumours. Although statistically significant, this correlation was not always very strong (tumour no 1, $\beta$ = 0.44), which suggests that other factors might also play a role.

Although introduction of 'cut-off-values' for the parameters that were investigated is arbitrary, there seems to be a clear relationship between the number of fulfilled conditions and [$^{131}$I]cG250 uptake, indicating that when certain conditions are present in a tumour sample – antigen G250 expression, vascularity and percentage viable tumour cells above a certain threshold – higher

© Cancer Research Campaign 1998 

**Table 3** Influence of the parameters on [$^{131}$I]-cG250 uptake per patient expressed as standardized regression coefficients (beta) and corresponding significance level

| Tumour no. | n | cG250 expression | | RCK-102 expression | | PAL-E expression | | CD-31 expression | | Vascularity (PAL-E + CD-31) | |
|---|---|---|---|---|---|---|---|---|---|---|---|
| | | β | P | β | P | β | P | β | P | β | P |
| 1 | 40 | 0.44 | * | 0.36 | ‡ | 0.01 | ‡ | −0.27 | ‡ | −0.26 | ‡ |
| 2 | 31 | −0.22 | ‡ | 0.80 | ** | −0.22 | ‡ | −0.22 | ‡ | −0.39 | * |
| 3 | 36 | 0.74 | ** | 0.07 | ‡ | −0.11 | ‡ | 0.31 | † | 0.19 | ‡ |
| 4 | 33 | 0.69 | ** | 0.26 | * | 0.10 | ‡ | −0.19 | † | 0.01 | ‡ |

*n*, number of tumour samples. *P* = significance level: ‡$P > 0.10$, †$0.05 < P < 0.10$, *$0.01 < P < 0.05$, ** $P < 0.01$.

**Table 4** Pearson correlation coefficients (*r*) between median (p50) [$^{131}$I]-cG250 uptake and the number of fulfilled conditions

| Tumour no | n | No. of fulfilled conditions | Median [$^{131}$I]cG250 uptake (p50) | r | P-value |
|---|---|---|---|---|---|
| | 13 | 0 | 0.0041 | | |
| 1 | 5 | 1 | 0.0058 | 0.39 | 0.01 |
| | 13 | 2 | 0.0086 | | |
| | 9 | 3 | 0.0136 | | |
| | 8 | 0 | 0.0022 | | |
| 2 | 11 | 1 | 0.0028 | 0.35 | 0.05 |
| | 11 | 2 | 0.0036 | | |
| | 1 | 3 | | | |
| | 0 | 0 | | | |
| 3 | 5 | 1 | 0.0041 | 0.47 | 0.004 |
| | 7 | 2 | 0.0044 | | |
| | 24 | 3 | 0.0060 | | |
| | 1 | 0 | | | |
| 4 | 9 | 1 | 0.0013 | 0.72 | 0.001 |
| | 12 | 2 | 0.0078 | | |
| | 11 | 3 | 0.0378 | | |

*n*, number of tumour samples.

[$^{131}$I]cG250 uptake occurs. This observation confirms that antibody uptake results from a combination of factors rather than from one factor alone.

There is compelling evidence from animal studies that high antigen expression of the recognized tumour-associated antigen is important for high antibody uptake: Shockley et al (1992) investigated accumulation of three anti-melanoma antibodies (MAbs 436, INDI and 9.2.27) in two different human melanoma xenografts in nude mice and concluded that antibody accumulation was primarily determined by antigen expression levels, although other physiological parameters, e.g. vascular permeability and vascular volume, could not be excluded. One of the few studies elegantly showing that enhanced antigen expression can result in enhanced antibody uptake was performed by Greiner et al (1987): these investigators were able to show that up-regulation of the tumour-associated antigen TAG72 by human interferon alpha (IFN-α) treatment was accompanied by an increase in MAb B72.3 uptake. They concluded that IFN-α treatment might be a modality to overcome antigenic heterogeneity and enhance the effect of monoclonal antibody therapy. Similarly, Wilder et al (1993) found that increased antigen expression induced by hyperthermia was associated with augmented MAb NR-LU-10 uptake in s.c. HCT-8 human colonic adenocarcinoma xenografts in nude mice.

Detailed investigations in patients in which factors determining antibody uptake are studied are rare, and the few results of clinical studies are contradictory: Buist et al (1995) investigated the relation between a number of tumour characteristics and uptake of the MAbs OV-TL 3 and chimeric MOv18 in tumour samples of patients with ovarian carcinoma. A close correlation between antibody uptake and antigen expression was shown, whereas no relation was found with tumour size, histological classification or percentage of cancer cells. Murray et al (1995) studied the effect of enhanced TAG-72 antigen expression following IFN-α treatment in relation to tumour uptake of MAb CC 49 in metastatic breast cancer patients. Although IFN-α treatment significantly up-regulated TAG-72 expression, there was no significant increase in antibody uptake, suggesting that other factors limited antibody uptake in this setting.

Jain et al (1990) have postulated a number of physiological barriers that might contribute to poor tumour localization of antibodies: heterogenous blood supply, (locally) elevated interstitial fluid pressure and large transport distances in the interstitium. Based on a mathematical model it has been postulated that binding of the antibody to antigen might reduce the diffusion rate of the antibody, i.e. the hypothesized 'binding barrier' (Fujimori et al, 1990). Because of macro- and microscopic tumour heterogeneity

© Cancer Research Campaign 1998

the influence of the factors mentioned above may vary from one location to another in the same tumour and from one time point to the next (Jain, 1990). In an earlier clinical study with murine MAb G250 (mG250) no apparent relation was found between the intratumoral distribution of [$^{131}$I]mG250, injected 8 days before surgery, and the distribution of [$^{99m}$Tc]human serum albumin (reflecting tumour perfusion), injected a few hours before surgery (Oosterwijk et al, 1993).

Morphologically, RCCs have a well established vascular bed, which is supposedly more leaky to macromolecules than the vasculature of normal tissue. This does not imply that all tumour regions are well perfused. Assuming that antibody uptake is facilitated by active perfusion and a concentration gradient, a well-established vascular bed alone is not sufficient for appropriate, homogeneous antibody targeting of tumours. Boucher et al (1996) described that increased interstitial fluid pressure was a direct result of the angiogenesis within a tumour. Thus, when enhanced interstitial fluid pressure limits antibody uptake, it seems likely that areas with abundant neovascularization would be poorly accessible to antibody. However, in the present study no correlation was found between [$^{131}$I]cG250 uptake and CD-31 expression or PAL-E expression. Nevertheless, this study showed in all tumours that a certain amount of vascularization (a fulfilled condition) correlates significantly with [$^{131}$I]cG250 uptake when other necessary conditions (e.g. certain antigen expression) are also fulfilled. Apparently enhanced interstitial fluid pressure in tumour tissue seems to play a less important role in MAb uptake than might be expected.

In conclusion, the present study indicated that high G250 antigen expression is a prerequisite, and probably the main driving force, for high cG250 antibody uptake. However, the fulfilment of other conditions, e.g. a certain amount of vascularization, also seems necessary for the establishment of high antibody uptake.

### REFERENCES

Blumenthal RD, Sharkey RM, Kashi R, Natale AM and Goldenberg DM (1992) Physiological factors influencing radioantibody uptake: a study of four human colonic carcinomas. *Int J Cancer* **51**: 935–941

Boucher Y, Leunig M and Jain RK (1996) Tumor angiogenesis and interstitial hypertension. *Cancer Res* **56**: 4264–4266

Buist MR, Kenemans P, Molthoff CF, Roos JC, den Hollander W, Brinkhuis M and Baak JP (1995) Tumor uptake of intravenously administered radiolabeled antibodies in ovarian carcinoma patients in relation to antigen expression and other tumor characteristics. *Int J Cancer* **64**: 92–98

Delisser HM, Newman PJ and Albelda SM (1993) Platelet endothelial cell adhesion molecule (CD31). *Curr Top Microbiol Immunol* **184**: 37–45

Fujimori K, Covell DG, Fletcher JE and Weinstein JN (1990) A modeling analysis of monoclonal antibody percolation through tumors: a binding-site barrier. *J Nucl Med* **31**: 1191–1198

Greiner JW, Hand PH, Colcher D, Weeks M, Thor A, Noguchi P, Pestka S and Schlom J (1987) Modulation of human tumor antigen expression. *J Lab Clin Med* **109**: 244–261

Jain RK (1990) Physiological barriers to delivery of monoclonal antibodies and other macromolecules in tumors. *Cancer Res* **50**: 814s–819s

Juweid M, Sharkey RM, Markowitz A, Behr T, Swayne LC, Dunn R, Hansen HJ, Shevitz J, Leung SO, Rubin AD, Herskovic T, Hanley D and Goldenberg DM (1995) Treatment of non-Hodgkin's lymphoma with radiolabeled murine, chimeric, or humanized LL2, an anti-CD22 monoclonal antibody. *Cancer Res* **55**: 5899s–5907s

Kaminski MS, Zasadny KR, Francis IR, Fenner MC, Ross CW, Milik AW, Estes J, Tuck M, Regan D, Fisher S, Glenn SD and Wahl RL (1996) Iodine-131-anti-B1 radioimmunotherapy for B-cell lymphoma. *J Clin Oncol* **14**: 1974–1981

Murray JL, Macey DJ, Grant EJ, Rosenblum MG, Kasi LP, Zhang HZ, Katz RL, Riger PT, Lebherz D, Bhadkamkar V, Greiner JW, Schlomm J and Podoloff DA (1995) Enhanced TAG-72 expression and tumor uptake of radiolabeled monoclonal antibody CC49 in metastatic breast cancer patients following alpha-interferon treatment. *Cancer Res* **55**: 5925s–5928s

Oosterwijk E, Ruiter DJ, Hoedemaker PJ, Pauwels EK, Jonas U, Zwartendijk J and Warnaar SO (1986) Monoclonal antibody G250 recognizes a determinant present in renal cell carcinoma and absent from normal kidney. *Int J Cancer* **38**: 489–494

Oosterwijk E, Bander NH, Divgi CR, Welt S, Wakka JC, Finn RD, Carswell EA, Larson SM, Warnaar SO, Fleuren GJ, Oettgen HF and Old LJ (1993) Antibody localization in human renal cell carcinoma: a phase I study of monoclonal antibody G250. *J Clin Oncol* **11**: 738–750

Press OW, Eary JF, Appelbaum FR, Martin PJ, Nelp WB, Glenn S, Fisher DR, Porter B, Matthews DC, Gooley T and Bernstein ID (1995) A phase II trial of 131I-B1 (anti-CD20) antibody therapy with autologous stem cell transplantation for relapsed B cell lymphomas. *Lancet* **346**: 336–340

Ramaekers F, van Niekerk C, Poels L, Schaafsma E, Huijsmans A, Robben H, Schaart G and Vooijs P (1990) Use of monoclonal antibodies to keratin 7 in the differential diagnosis of adenocarcinomas. *Am J Pathol* **136**: 641–655

Schlingemann RO, Dingjan GM, Emeis JJ, Blok J, Warnaar SO and Ruiter DJ (1985) Monoclonal antibody PAL-E specific for endothelium. *Lab Invest* **52**: 71–76

Shockley TR, Lin K, Sung C, Nagy JA, Tompkins RG, Dedrick RL, Dvorak HF and Yarmush ML (1992) A quantitative analysis of tumor specific monoclonal antibody uptake by human melanoma xenografts: effects of antibody immunological properties and tumor antigen expression levels. *Cancer Res* **52**: 357–366

Steffens MG, Boerman OC, Oosterwijk-Wakka JC, Witjes JA, Koenders EB, Oyen WJG, Buijs WCAM, Debruyne FMJ, Corstens FHM and Oosterwijk E (1997) Targeting of renal cell carcinoma with iodine-131 labeled chimeric monoclonal antibody G250. *J Clin Oncol* **15**: 1529–1537

Wilder RB, Langmuir VK, Mendonca HL, Goris ML and Knox SJ (1993) Local hyperthermia and SR 4233 enhance the antitumor effects of radioimmunotherapy in nude mice with human colonic adenocarcinoma xenografts. *Cancer Res* **53**: 3022–3027

Wilder RB, Denardo GL and Denardo SJ (1996) Radioimmunotherapy: recent results and future directions. *J Clin Oncol* **14**: 1383–1400

© Cancer Research Campaign 1998